# Zoonotic Vectorborne Pathogens and Ectoparasites of Dogs and Cats in Eastern and Southeast Asia

**DOI:** 10.3201/eid2606.191832

**Published:** 2020-06

**Authors:** Vito Colella, Viet L. Nguyen, Do Y. Tan, Na Lu, Fang Fang, Yin Zhijuan, Jiangwei Wang, Xin Liu, Xinghui Chen, Junyan Dong, Wisnu Nurcahyo, Upik K. Hadi, Virginia Venturina, Kenneth B.Y. Tong, Yi-Lun Tsai, Piyanan Taweethavonsawat, Saruda Tiwananthagorn, Thong Q. Le, Khanh L. Bui, Malaika Watanabe, Puteri A.M.A. Rani, Giada Annoscia, Frédéric Beugnet, Domenico Otranto, Lénaïg Halos

**Affiliations:** University of Melbourne, Melbourne, Victoria, Australia (V. Colella);; University of Bari, Bari, Italy (V. Colella, V.L. Nguyen, G. Annoscia, D. Otranto);; Boehringer Ingelheim Animal Health, Lyon, France (D.Y. Tan, Na Lu, F. Beugnet, L. Halos);; Guangxi University, Nanning, China (F. Fang); KangBao Pet Hospital, Guilin, China (Y. Zhijuan);; Sapphire Veterinary Hospital, Shanghai, China (J. Wang);; Meilianzhonghe Veterinary Referral Center, Beijing, China (X. Liu);; Chongyisheng Veterinary Hospital, Chengdu, China (X. Chen);; Nanjing Police Dog Research Institute, Nanjing, China (J. Dong);; Gadjah Mada University, Yogyakata, Indonesia (W. Nurcahyo);; Bogor University Indonesia, Jakarta, Indonesia (U.K. Hadi);; Central Luzon State University, Nueva Ecija, Philippines (V. Venturina);; Animal & Avian Veterinary Clinic, Yishun, Singapore (K.B.Y. Tong);; National Pingtung University of Science and Technology, Pingtung, Taiwan (Y.L. Tsai);; Chulalongkorn University, Bangkok, Thailand (P. Taweethavonsawat);; Chiang Mai University, Chiang Mai, Thailand (S. Tiwananthagorn);; Nong Lam University, Ho Chi Minh City, Vietnam (T.Q. Le);; Vietnam National University of Agriculture, Hanoi, Vietnam (K.L. Bui);; University Putra Malaysia, Selangor, Malaysia (M. Watanabe, P.A.M.A. Rani)

**Keywords:** Zoonoses, vector-borne diseases, vector-borne infections, companion animals, dogs, cats, eastern Asia, Southeast Asia, ectoparasites, pathogens, vectors, parasites

## Abstract

To provide data that can be used to inform treatment and prevention strategies for zoonotic pathogens in animal and human populations, we assessed the occurrence of zoonotic pathogens and their vectors on 2,381 client-owned dogs and cats living in metropolitan areas of 8 countries in eastern and Southeast Asia during 2017–2018. Overall exposure to ectoparasites was 42.4% in dogs and 31.3% in cats. Our data cover a wide geographic distribution of several pathogens, including *Leishmania infantum* and zoonotic species of filariae, and of animals infested with arthropods known to be vectors of zoonotic pathogens. Because dogs and cats share a common environment with humans, they are likely to be key reservoirs of pathogens that infect persons in the same environment. These results will help epidemiologists and policy makers provide tailored recommendations for future surveillance and prevention strategies.

Asia is the largest continent in the world, known for its thriving biocultural diversity. Today, countries in Asia are experiencing a rapid social, demographic, and economic transformation, thereby placing this region as an ever-growing economic powerhouse in the years to come. Sustained economic growth in Asia has resulted in increased demand for products and services and substantial urbanization (*1*). These factors have triggered a series of human-mediated environmental alterations, such as deforestation and encroachment of humans into natural ecosystems, that now link previously isolated ecologic niches and give pathogens new opportunities to thrive (*2*). During the past century, Asia has been in the limelight for emergence and pathogenicity of a large number of infectious diseases that have taken a substantial toll on the health of millions of persons (*1*). Striking examples include the emergence of severe acute respiratory syndrome, infections with the highly pathogenic avian influenza A(H5N1) virus, and coronavirus disease (COVID-19). Recently, human modification of natural habitats resulted in the emergence of a tick vector of Kyasanur Forest disease virus, a zoonotic vectorborne flavivirus that causes severe hemorrhagic fever with a fatality rate of 3%–10% (*3*).

Also implicated in the changing epidemiology of pathogens of public health concern in eastern and Southeast Asia are dogs and cats (*4*–*6*). In remote areas of eastern and Southeast Asia, three quarters of dogs are classified as stray or community dogs (*7*). Increases in living standards have led to a dramatic surge in the number of pet dogs and cats living in metropolitan settings (*8*,*9*). In China, the population of pet dogs is estimated to grow by 5 million per year. Along with this increase in companion animal ownership, the risk of acquiring parasitic zoonoses from companion dogs and cats represents an ongoing, yet neglected, threat (*10*,*11*).

Implementation of effective measures to control zoonotic diseases must rely on the elucidation of pathogens and reservoir hosts in a given area. For most countries in Asia, limited knowledge about the agents parasitizing dogs and cats, including those transmissible to humans, hinders the establishment of proper strategies for treatment and prevention of zoonotic pathogens in animal and human populations. Although previous investigations have explored the occurrence of zoonotic diseases in animals living in remote areas (*4**–**7*), our year-long multicenter study explored the occurrence of vectorborne pathogens and ectoparasites in pet dogs and cats from metropolitan areas in eastern and Southeast Asia.

## Methods

Our study involved academic institutions and private facilities of eastern Asia (China and Taiwan) and Southeast Asia (Indonesia, Malaysia, the Philippines, Singapore, Thailand, and Vietnam). To provide capacity building and compliance with the study procedures, trainings were performed at local institutions as needed. The protocol of this study was approved by the Ethics Committee of the Department of Veterinary Medicine, University of Bari (protocol no. 13/17). At partner institutions, animal owners read, approved, and signed an owner informed consent, which contained information about study procedures.

During 2017–2018, local investigators sampled 10 client-owned dogs and 10 client-owned cats each month for 12 months in each country, except China, where 40 dogs and 40 cats each month were sampled. Inclusion criteria were a history of regular outdoor access and having not received recent antiparasitic treatments. Data on the animals’ location, age, breed, and sex were recorded. 

### Veterinary Examination

Veterinarians performed a complete examination of the animals, reporting abnormalities in rectal temperature, overall physical condition, demeanor, nasal discharge, skin/haircoat, eyes, superficial lymph nodes, respiratory system (breathing), cardiovascular system (mucous membranes), and fecal consistency. The examinations included checking for the presence of ectoparasites (ticks, fleas, lice, and mites) by examining the whole-body surface for >5 minutes. The veterinarians inspected both eyes, including a thorough examination under the third eyelid to detect adult *Thelazia callipaeda* eyeworms. They also performed testing for lesions evocative of sarcoptic mange or demodicosis (deep skin scraping), cheyletiellosis (tape test), or otoacariosis (earwax examination). 

Sampled parasites were stored in vials containing 70% ethanol and sent for morphologic and molecular identification at the University of Bari (Bari, Italy), where we examined adult and nymph ticks under a stereomicroscope. We clarified tick larvae, fleas, lice, and fur mites in 10% potassium hydroxide overnight, mounted in Hoyer’s medium and observed under an optical microscope (*12*). We used morphologic keys to identify all ectoparasites to the species level (*13**–**20*). For mite identification, we minced crusty skin lesions by using disposable surgical blades, added drops of saline solution on a glass slide, observed the slides under an optical microscope, and identified the mites according to morphologic appearance (*18*,*21*). We mounted anterior and posterior extremities of adult *T. callipaeda* eyeworms in lactophenol and identified them (*22*).

### Molecular Identification of Ectoparasites

To confirm morphologic identifications of ectoparasite species, we subjected a representative subpopulation (≈20%) of the ectoparasites to DNA extraction and amplification of target genes (Appendix Table). For ticks, fleas and lice, we isolated genomic DNA (gDNA) by using the DNeasy Blood and Tissue Kit (QIAGEN, https://www.qiagen.com) according to the manufacturer’s instructions. We isolated gDNA from a small portion of the idiosoma of ticks (*23*) and from the anterior dorsal part of the abdomen of fleas (*24*). We selected individual lice and mites under an optical microscope and extracted gDNA by using a QIAamp DNA Micro Kit (QIAGEN).

### Blood Collection and Processing

From each study animal, we collected ≈2 mL of blood in a tube with anticoagulant and processed it as follows. For dogs, we used an aliquot of the blood sample for the ELISA-based technology SNAP 4Dx Plus test (IDEXX Laboratories, Inc., https://www.idexx.com) to detect *Dirofilaria immitis* antigen and antibodies against *Anaplasma phagocytophilum*/*A. platys*, *Borrelia burgdorferi* sensu lato, and *Ehrlichia canis*/*E. ewingii* as described and the SNAP *Leishmania* test (IDEXX Laboratories, Inc.) to detect antibodies against *Leishmania infantum/L. donovani* as described. For cats, we used an aliquot of blood to detect antigens of feline leukemia virus (FeLV) and *D. immitis* and antibodies against feline immunodeficiency virus (FIV). We used the SNAP Combo FIV/FeLV and SNAP Heartworm RT Test (IDEXX Laboratories, Inc.). For dogs and cats, we blotted 2 spots of blood (125 μL each, total 250 μL/animal) onto Whatman FTA cards (Sigma-Aldrich Corp., https://www.sigmaaldrich.com), stored the cards overnight (>6 h) at room temperature for blood to dry, and put them in a zip-locked plastic bag. 

### DNA Extraction, Amplification, Purification, and Sequencing

From each Whatman FTA card, we punched out 5 disks of 3.0-mm each (Uni-Core 150 punch; GE Healthcare, https://www.gelifesciences.com) and placed them in each well of a 96-well plate (QIAcube HT kit Plasticware; QIAGEN) and included a negative control (Whatman FTA card blotted with dog blood naive to the pathogens in this study) for each plate. Subsequently, we added a 200-μL solution (180 μL of buffer ATL and 20 μL of proteinase K) to each well and subjected samples to prelysate overnight incubation at 56°C in a 711 CT incubator (Asal s.r.l., http://www.asal.it). We extracted DNA by using a QIAcube HT and the QIAamp 96 DNA QIAcube HT kit (QIAGEN) according to manufacturer instructions. We tested all gDNA isolated from dried blood samples by conventional PCR (cPCR) (Appendix Table). We detected *Leishmania* protozoa by quantitative PCR (qPCR) and further tested only samples scoring positive in duplicates by qPCR by cPCR on the internal transcribed spacer 2 region and kinetoplast DNA for species identification (Appendix Table).

For all PCRs, we included positive controls (DNA of pathogen-positive blood samples) and negative controls (DNA of pathogen-negative blood samples). We visualized PCR amplicons from nematodes and apicomplexan protozoa by capillary electrophoresis by using a QIAxcel DNA screening gel cartridge on a QIAxcel system (QIAGEN for each) and used a QX DNA Size Marker (QIAGEN) to size PCR products. We injected a QX Alignment Marker (QIAGEN), which consisted of 15-bp and 3,000-bp fragments, onto the cartridge with each sample. We then determined the PCR product sizes by using QIAxcel Screen Gel 1.4.0 software (QIAGEN). We subjected cPCR products from *Leishmania* spp. protozoa, *Thelazia* spp. eyeworms, ticks, fleas, lice, and mites to electrophoresis in a 2% agarose gel stained with Gel Red (VWR International PBI, https://it.vwr.com) and visualized them on a Kodak Gel Logic 100 gel documentation system (https://www.kodak.com).

We purified all cPCR amplicons obtained and sequenced them in both directions in an automated sequencer ABI-PRISM 377 (ThermoFisher Scientific, https://www.thermofisher.com). We edited and aligned the sequences by using Geneious Prime software (https://www.geneious.com) and compared them with each other and with those available in the GenBank database by using BLAST (http://blast.ncbi.nlm.nih.gov/Blast.cgi).

### Statistical Analyses

We calculated frequency values as the proportion of positive animals to the total number of examined animals and the relative frequency of occurrence of each species of parasite as the proportion of animals infested by a given parasite species/group within the total number of positive results within a given parasite species/group. We calculated 95% CIs by using the Wilson score interval.

We categorized animals into 3 age groups (<1, >1 to <5, and >5 years). We used the χ^2^ test to investigate associations between parasitic infection/exposure or infestation by ectoparasites and age group or clinical observations. We analyzed the Cohen κ coefficient and dependent and independent variables by using GraphPad Prism 8 (http://www.graphpad.com). We considered p<0.01 to indicate significance.

## Results

**Figure 1:**
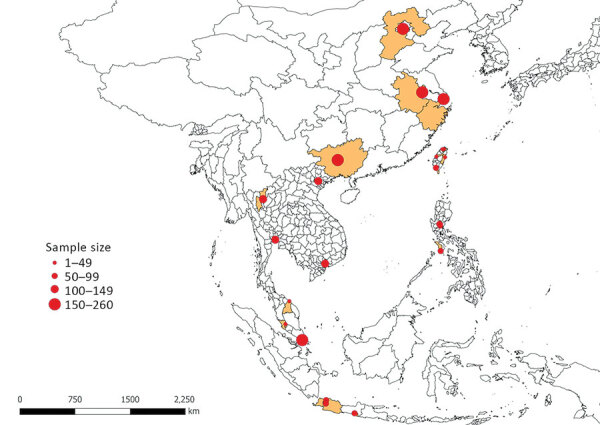
Geographic distribution and size of dog and cat samples in study of ectoparasites and vectorborne zoonotic pathogens of dogs and cats in Asia, 2017–2018. Highlighted areas represent the geographic regions from which samples were collected in China, Indonesia, Malaysia, the Philippines, Singapore, Taiwan, Thailand, and Vietnam.

Our study sample consisted of 2,381 animals (1,229 dogs and 1,152 cats). Samples were collected from animals living in 23 main cities (and neighboring localities) in 8 countries in Asia, specifically China (Beijing, Nanjing, Shanghai, and Guangxi Province), Taiwan (Taipei, Taoyuan, Changhua, Pingtung, and Hualien), Indonesia (Jakarta, Bogor, and Yogyakarta), Malaysia (Klang Valley region and Kota Bharu), the Philippines (Cabanatuan, San Jose, and Munoz), Singapore, Thailand (Bangkok and Chiang Mai), and Vietnam (Hanoi and Ho Chi Minh City) (Figure 1).

The dog population was composed of 565 (46.0%) females, 660 (53.7%) males, and 4 (0.3%) with unreported data; the cat population was composed of 543 (47.1%) females, 606 (52.6%) males, and 3 (0.3%) with unreported data. Ages of dogs ranged from 2 months to 20 years (mean 5.1 years, median 4.0 years), and ages of cats ranged from 2 months to 20 years (mean 2.7 years, median 2.0 years) (Table 1).

**Table 1:** Distribution of 1,229 dogs and 1,152 cats from select countries, by age group, husbandry, and sex, in study of ectoparasites and vectorborne zoonotic pathogens of dogs and cats, Asia, 2017–2018

Country (total no. animals; no. dogs, no. cats)	Age, y									Husbandry						Sex				
	<1			>1 to <5			>5			Urban area			Rural area			M			F	
	Dogs	Cats		Dogs	Cats		Dogs	Cats		Dogs	Cats		Dogs	Cats		Dogs	Cats		Dogs	Cats
China (971; 481, 490)*	122	228		182			172	69		464	484		17	6		289	273		192	217
Indonesia (173; 95, 78)	30	22		50	46		15	10		68	63		27	15		51	39		44	39
Malaysia (91; 45, 46)	9	30		11	14		25	2		45	1		0	45		20	27		25	19
Philippines (235; 120, 115)	45	50		50	59		20	5		96	91		24	23		51	60		68	54
Singapore (245; 116, 129)	3	61		14	26		98	42		115	128		1	1		50	67		63	60
Taiwan (186; 132, 54)	10	26		45	20		77	8		81	35		51	19		62	28		69	26
Thailand (240; 120, 120)	15	35		49	66		54	19		89	101		31	19		69	49		51	71
Vietnam (240; 120, 120)	48	80		49	27		20	0		81	86		39	19		66	63		53	57
All (2,381; 1,229, 1,152)	282	532		450	451		481	155		1,039	989		190	147		658	606		565	543

*Beijing (240; 119, 121); Nanjing (240; 120, 120); Shanghai (240; 120, 120); Guangxi Province (251; 122, 129).

**Figure 2:**
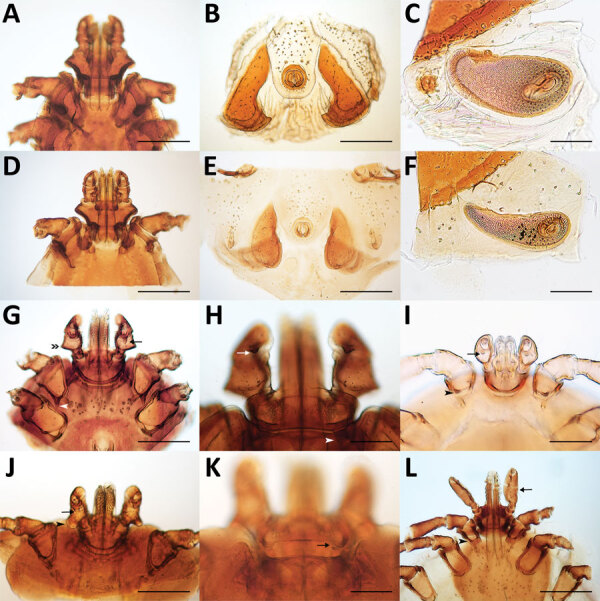
Morphologic characteristics of ticks collected from dogs and cats in study of ectoparasites and vectorborne zoonotic pathogens of dogs and cats in Asia, 2017–2018. A–C) Male *Rhipicephalus haemaphysaloides* tick with hexagonal basis capitulum (A); typical sickle-shape adanal plates (B); and spiracular plate with comma shape, broad throughout its length (C). D–F) Male *R. sanguineus* tick with hexagonal basis capitulum (D); subtriangular adanal plates (E); and comma-shaped spiracular plate, elongated throughout its length (F). G) Female *Haemaphysalis longicornis* tick with enlarged lateral palp article II (double arrowhead), ventral spur on palp article III (arrow), and internal spur on coxa I (white arrowhead), relatively long and pointed. H) *H. longicornis* tick palp article III with retrograde dorsal spur (arrow) and cornua one third the length of the basis capitulum (arrowhead). I) Larva of *Haemaphysalis wellingtoni* palp article II slightly broader than article III, internal spur on coxa I (arrowhead) and strong and sharp ventral spur on palpal article III (arrow). J–K) Female *Haemaphysalis campanulata* tick with well-defined ventral spur (arrow) on palp article III, palp article II strongly salient laterally, with flared and bell-shaped posterior margin (arrowhead) (J) and with short cornua (arrow) (K). L) *Ixodes* sp. female tick with long palp (arrow) and short spur on coxa I (arrowhead). Scale bars in panels A, B, D, E, G, J, and L indicate 500 μm; scale bars in panels C, F, H, and K indicate 200 μm; scale bar in panel I indicates 100 μm.

**Figure 3:**
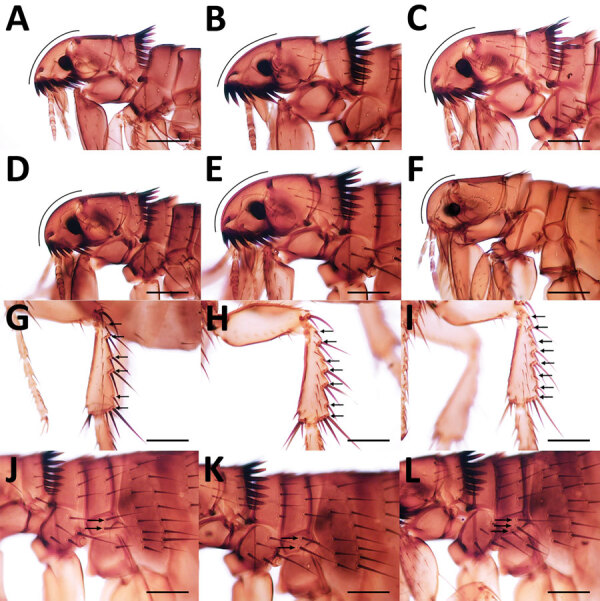
Morphologic characteristics of fleas collected from dogs and cats in study of ectoparasites and vectorborne zoonotic pathogens of dogs and cats in Asia, 2017–2018. A) *Ctenocephalides felis* male flea head; B) *C. felis* female flea head showing acute anterior margin; C) *C. canis* male flea; D) *Ctenocephalides orientis* male flea; E) *C. orientis* female flea; F) *Xenopsylla cheopis* male flea with strongly rounded anterior margin and absence of ctenidia; G) *C. felis* flea with 6 setae-bearing notches (arrows) on the dorsal margin of the hind tibia; H) *C. orientis* flea with 7 setae-bearing notches; I) *C. canis* flea with 8 setae-bearing notches; J) *C. felis* flea with 2 setae on the lateral metonotal area (arrows); K) *C. orientis* flea with 2 setae; L) *C. canis* fleas with 3 setae. Scale bars indicate 200 μm.

**Figure 4:**
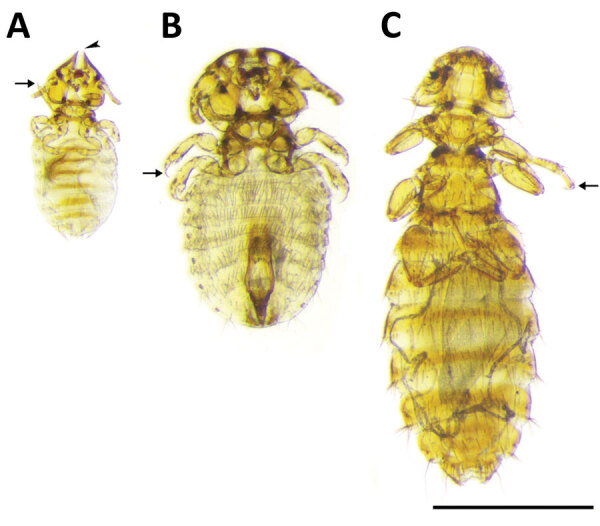
Chewing lice collected from dogs and cats in study of ectoparasites and vectorborne zoonotic pathogens of dogs and cats in Asia, 2017–2018. A) Female *Felicola subrostratus* louse with triangular head and pointed anteriorly. The median longitudinal groove (arrowhead) on the head fits around the shaft of the hair of the host. Thorax is short and legs are small, ending with a single claw (arrow). B) *Trichodectes canis* male louse with short thorax, flattened head with quadrangular shape, broader than long; each leg with only 1 claw on tarsus (arrow). C) *Heterodoxus spiniger* female louse with subtriangular head, rounded anteriorly. The thorax is considerably longer than wide. Each leg has 2 claws on the tarsus (arrow). Scale bar indicates 1 mm.

**Figure 5:**
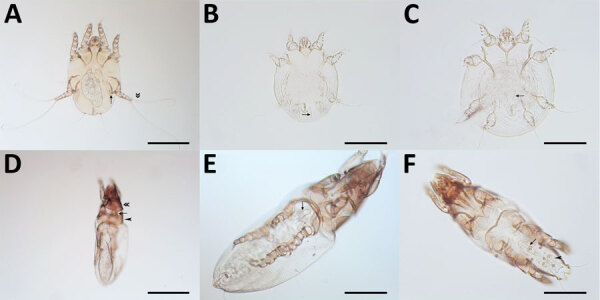
Mites collected from dogs and cats in study of ectoparasites and vectorborne zoonotic pathogens of dogs and cats in Asia, 2017–2018. A) *Otodectes cynotis* female mite with greatly reduced last pair of legs (arrow); the third pair of legs terminates in 2 long and whip-like setae (double arrowhead). B) *Sarcoptes scabiei* male mite with strong and spine-like dorsal setae (arrow). C) *Notoedres cati* mite with narrow and not spine-like setae. D) Female *Lynxacarus radovskyi* cat fur mite with cylindrical and heavily striated idiosoma, well-developed head plate (double arrowhead) with convex posterior margin; propodosomal plate (arrowhead) with posterior margin broadly rounded, connected mediodorsally to head plate by a narrow-sclerotized band (arrow). E) *L. radovskyi* female mite genital apparatus (arrow) positioned between coxae III in female. F) *L. radovskyi* male mite with genital apparatus (arrow) positioned between coxae IV (arrow) and circular genital discs (arrowhead). Scale bars in panels A and D indicate 200 μm; scale bars in panels B, C, E, and F indicate 100 μm.

**Figure 6:**
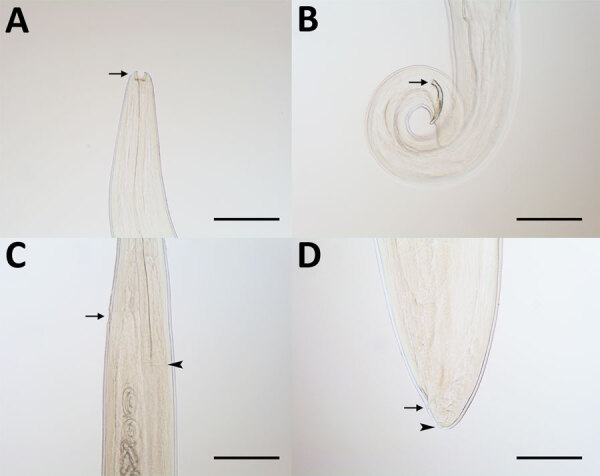
*Thelazia callipaeda* eyeworms collected from animals in China in study of ectoparasites and vectorborne zoonotic pathogens of dogs and cats in Asia, 2017–2018. A) *T. callipaeda* male eyeworm with buccal capsule (arrow); B) posterior end of male eyeworm with short and crescent-shaped spicule (arrow); C) anterior portion of *T. callipaeda* female eyeworm with vulva (arrow) located posterior to the esophagus-intestinal junction (arrowhead); and D) posterior end of female eyeworm showing anus (arrow) and phasmids (arrowhead). Scale bars indicate 200 μm.

Overall, 42.4% (95% CI 39.7%–45.2%) of dogs and 31.3% (95% CI 28.4%–33.7%) of cats had >1 ectoparasite detected, exposure to vectorborne parasites, or both (Tables 2,3,4,5,6; Figures 2,3,4,5,6). In particular, 33.5% (95% CI 30.9%–36.2%) of dogs and 31.3% (95% CI 28.4%–34.0%) of cats were infested with >1 ectoparasite, and 22.8% (95% CI 20.5%–25.2%) of dogs and 0.5% (95% CI 0.2%–1.1%) of cats were detected with or exposed to >1 vectorborne parasite. *T. callipaeda* eyeworms were detected in 1.7% (95% CI 0.8%–3.3%) of dogs from China, specifically in 6.7% (95% CI 3.4–12.7) of dogs and 0.6% (95% CI 0.2%–1.8%) of cats from Beijing.

**Table 2:** Frequency of tick, flea, and lice detection on 1,229 dogs and 1,152 cats, by country, in study of ectoparasites and vectorborne zoonotic pathogens of dogs and cats, Asia, 2017–2018

Country (no. dogs, no. cats)	Detection frequency, % (95% CI)							
	Ticks			Fleas			Lice	
Dogs	Cats	Dogs	Cats	Dogs	Cats
China (481, 490)	6.0 (4.2–8.5)	0.2 (0.0–1.1)		3.1 (1.9–5.1)	4.5 (3.0–6.7)		0.6 (0.2–1.8)	0.8 (0.0–2.0)
Indonesia (95, 78)	42.1 (32.7–52.2)	10.3 (5.3–18.9)		16.8 (10.6–25.6)	53.9 (42.9–64.5)		6.3 (2.9–13.1)	33.3 (23.9–44.4)
Malaysia (45, 46)	4.4 (1.2–14.8)	0		4.4 (1.2–14.8)	89.1 (77.0–95.3)		0	8.7 (3.4–20.3)
Philippines (120, 115)	67.5 (58.7–75.2)	24.4 (17.4–32.9)		80.0 (72.0–86.2)	54.8 (45.7–63.6)		52.5 (43.6–61.2)	26.1 (18.9–34.8)
Singapore (116, 129)	8.6 (4.7–15.1)	1.6 (0.4–5.5)		0.9 (0.0–4.7)	3.9 (1.7–8.7)		0.9 (0.0–4.7)	3.9 (1.7–8.7)
Taiwan (132, 54)	12.9 (8.2–19.7)	5.6 (1.9–15.1)		9.1 (5.3–15.2)	20.4 (11.8–32.9)		0.8 (0.0–4.2)	1.9 (0.3–9.7)
Thailand (120, 120)	27.5 (20.3–36.1)	0		20.8 (14.5–28.9)	19.2 (13.1–27.1)		2.5 (0.8–7.1)	0
Vietnam (120, 120)	51.7 (42.8–60.4)	0.8 (0.1–4.6)		12.5 (7.7–19.6)	15.8 (10.4–23.4)		0.8 (0–4.6)	0
All (1,229, 1152)	22.3 (20.1−24.7)	3.7 (2.8–5.0)		14.8 (12.9–16.9)	19.6 (17.4–22.0)		6.4 (5.1–7.8)	6.1 (4.8–7.6)

**Table 3:** Frequency of mite detection on 1,229 dogs and 1,152 cats in study of ectoparasites and vectorborne zoonotic pathogens of dogs and cats, Asia, 2017–2018

Country (no. dogs, no. cats)	Detection frequency, % (95% CI)								
	Scabies mites								*Lynxacarus radovskyi*, cats
	*Sarcoptes scabiei*, dogs	*Notoedres cati*, cats		*Demodex* spp.			*Otodectes cynotis*		
				Dogs	Cats		Dogs	Cats	
China (481, 490)	0.6 (0.2–1.8)	0.2 (0−1.1)		0.6 (0.2–1.8)	0.2 (0−1.1)		1.3 (0.6–2.7)	5.1 (3.5–7.4)	0
Indonesia (95, 78)	3.2 (1.1–8.9)	34.6 (25.0–45.7)		1.1 (0.2–5.7)	0		0	12.8 (7.1–22.0)	5.1 (2.0–12.5)
Malaysia (45, 46)	0	0		0	0		0	17.4 (9.09–30.72)	4.4 (1.2–1.45)
Philippines (120, 115)	0	0.9 (0.1–4.7)		3.3 (1.3–8.3)	0.9 (0.1–4.7)		0	2.6 (0.9–7.4)	0
Singapore (116, 129)	2.7 (0.5–6.1)	0		4.3 (1.8–9.7)	0		0.9 (0.1–4.7)	19.4 (13.5–27.0)	34.9 (27.2–43.4)
Taiwan (132, 54)	0	0		0.2 (0.04–5.4)	0		2.3 (0.8–6.5)	7.4 (2.9–17.5)	0
Thailand (120, 120)	0	0		0	0		0	1.7 (0.0–5.8)	0
Vietnam (120, 120)	0.8 (0.1–4.6)	0		2.5 (0.8–7.1)	0		0.8 (0.1–4.6)	10 (5.8–16.7)	0
All (1,229, 1,152)	0.7 (0.4–1.4)	2.5 (1.7–3.4)		1.5 (0.9–2.3)	0.2 (0−0.6)		0.9 (0.5–1.6)	7.7 (6.3–9.4)	4.4 (3.4–5.6)

**Table 4:** Frequency of tick detection on 1,229 dogs and 1,152 cats and molecular identification of ticks, in study of ectoparasites and vectorborne zoonotic pathogens of dogs and cats, Asia, 2017–2018

Ectoparasite	Location			Relative frequency of occurrence, % (95% CI)		GenBank accession nos.
	Dogs	Cats		Dogs	Cats	
Tick						
*Rhipicephalus sanguineus*	All countries	Philippines, Singapore, Indonesia, Taiwan, China		96.6 (93.6–98.2)	95 (83.5–98.6)	MN685287–321, MT320104–5
*R. haemaphysaloides*		Taiwan		0.8 (0.2–2.7)		MN653239–40
*Haemaphysalis hystricis*	Thailand			0.4 (0–2.1)		MN658833
*H. wellingtoni*	Thailand, Indonesia			0.8 (0.2–2.7)		MN658820–1
* H. campanulata*	China			0.4 (0–2.1)		MN658817
* H. longicornis*	China	China		0.8 (0.2–2.7)	2.5% (0.4–12.9)	MN658797–800
* Ixodes* sp.		Taiwan			2.5 (0.4–12.9)	MT035959
Fleas						
* Ctenocephalides felis*	All	All		65.1 (57.7–71.8)	98.7 (95.5–99.6)	MT027205–8, MT027227, MT027230
* C. canis*	Vietnam, Philippines, Indonesia, China	Philippines		15.7 (22.0–21.9)	0.6 (0.1–3.5)	Not applicable
* C. orientis*	Vietnam, Thailand, Philippines, Singapore, Indonesia, Taiwan			19.2 (14.0–25.7)		MT027193–99
* Xenopsylla cheopis*		Indonesia			0.6 (0.1–3.5)	MT027228
Lice						
* Heterodoxus spiniger*	Vietnam, Thailand, Philippines, Taiwan, China			72.8 (63.5–80.5)		MT027225
* Trichodectes canis*	Philippines, Singapore, Indonesia			25.2 (17.8–34.4)		MT027226
* Felicola subrostratus*	Indonesia	Philippines, Indonesia, China		1.9 (0.5–6.8)	1.8 (0.3–9.3)	Not applicable
* Linognathus setosus*		Malaysia				Not applicable
Mites						
* Lynxacarus radovskyi*		Singapore, Malaysia, Indonesia		Not applicable	Not applicable	MN639734, MN639736

**Table 5:** Serologic and molecular detection of vectorborne pathogens in dogs in study of ectoparasites and vectorborne zoonotic pathogens of dogs and cats, Asia, 2017–2018*

Country	Detection frequency, %				
	*Ehrlichia canis*, Ab	*Anaplasma platys*, Ab	*Dirofilaria immitis*, Ag, PCR, Ag + PCR	*Hepatozoon canis*, PCR	*Babesia gibsoni*, PCR
Overall	14.8 (12.9–16.9)	7.1 (5.8–8.7)	3.5 (2.6–4.6), 2.3 (1.58–3.3), 3.9 (3.0–5.1)	1.6 (1.1–2.5)	1.0 (0.6–1.7)
China	1.9 (1–3.5)	0.2 (−1.2)	0	0.8 (0.3–2.1)	2.3 (1.3–4.0)
Indonesia	36.2 (27.2–46.2)	11.7 (6.7–19.7)	0	0	0
Malaysia	11.1 (4.8–23.5)	11.1 (4.8–23.5)	6.7 (2.3–17.7), 6.7 (2.3–17.7), 6.7 (2.3–17.9)	2.2 (0.4–11.6)	0
Philippines	33.0 (25.0–42.2)	17 (11.1–25.0)	17.9 (11.9–26.0), 13.6 (8.27–21.5), 29.8 (21.9–39.2)	9.7 (5.4–17.0)	0
Singapore	5.3 (2.4–11.0)	2.6 (0.9–7.4)	2.6 (0.9–7.4)	0	0.9 (0.1–4.7)
Taiwan	1.5 (0.42–5.4)	3 (1.2–7.5)	8.3 (4.7–14.3), 4.8 (2.07–10.8), 13.2 (8.0–21.0)	0	0
Thailand	45 (36.4–53.9)	24.2 (17.4–32.5)	4.2 (1.8–9.5), 4.2 (1.8–9.4), 5.8 (2.8–11.5)	3.3 (1.3–8.3)	0
Vietnam	25.8 (18.8–34.3)	13.3 (8.4–20.6)	0, 0.8 (0.1–4.6), 0.8 (0.1–4.6)	0.8 (0.1–4.6)	0

*Leishmania spp., *Borrelia* spp., and *Brugia* spp. not shown. Ab, antibodies; Ag, antigens; PCR samples positive by PCR-coupled Sanger sequencing.

**Table 6:** Molecular identification and GenBank accession numbers of vectorborne parasites detected in study of ectoparasites and vectorborne zoonotic pathogens of dogs and cats, Asia, 2017–2018

Parasite species	GenBank accession nos.
*Dirofilaria immitis* filariae	MT027229
*Brugia malayi* filariae	MT027200–1
*Brugia pahangi* filariae	MT027202–4
*Leishmania infantum* protozoa	MN699319–20
*Thelazia callipaeda* nematodes	MT040339–44
*Hepatozoon canis* protozoa	MN689651–71
*Babesia gibsoni* protozoa	MN689634–48

We detected co-infections with *Hepatozoon canis* and *D. immitis* heartworms in 4 dogs (2 each from Thailand and the Philippines). No tick infestations were found on dogs infected with *B. gibsoni*, but *Rhipicephalus sanguineus* ticks were detected on 50% of animals with *H. canis* infection. *H. canis* infection was correlated with infestation by *R. sanguineus* ticks (p = 0.0125).

A total of 4 dogs (0.3%, 95% CI 0.1%–0.8%) were positive for antibodies against *Leishmania infantum;* 2 of these dogs were from China (0.4%, 95% CI 0.1%–1.5%) and 1 each from Vietnam (0.8%, 95% CI 0.1%–4.6%) and the Philippines (0.9%, 95% CI 0.2%–5.0%). In addition, the 2 seropositive dogs from China were positive for *L. infantum* by qPCR and cPCR Sanger sequencing (Table 6).

A total of 2 dogs (0.2%, 95% CI 0–0.6) were positive for antibodies against *Borrelia burgdorferi* sensu lato. One was in the Philippines (0.9%, 95% CI 0.2%–4.9%), and 1 was in Indonesia (1.1%, 95% CI 0.2%–5.7%).

Overall, 5.2% (95% CI 4.1%–6.7%) of dogs were infected with filarial parasites according to antigen testing (3.5%, 95% CI 2.6%–4.6%), cPCR (2.7%, 95% CI 1.9%–3.7%), or both. cPCR-coupled sequencing identified *Brugia* spp. in 0.4% of dogs (95% CI 0.2%–0.9%) and in 15.2% (95% CI 6.6%–30.9%) of the samples positive for filariae by cPCR. Specifically, *B. pahangi* was found in dogs in Thailand (1.7%, 95% CI 0.5%–5.9%) and Malaysia (2.2%, 95% CI 0.4–11.6), and *B. malayi* was found in dogs in Vietnam (0.8%, 95% CI 0.1–4.6) and Thailand (0.8%, 95% CI 0.1%–4.6%). Using the kappa statistic, we found slight to fair agreement between antigen testing and cPCR (κ *=* 0.271, 95% CI 0.079–0.463) for the diagnosis of *D. immitis* in dogs. One cat (1.3%, 95% CI 0.2%–6.9%) from Indonesia was positive for *D. immitis* antigen.

*H. canis* infection was diagnosed for 1 cat from the Philippines (0.9%, 95% CI 0.2–5.1) and *B. gibsoni* for 3 cats in China (0.6%, 95% CI 0.2%–1.8%) and 1 cat in Singapore (0.8%, 95% CI 0.1–4.3). FIV antibodies were detected in 5.2% (95% CI 4.0%–6.6%) of cats and FeLV antigens in 2.9% (95% CI 2.1%–4.0%).

We compiled statistically significant associations for the detection of/exposure to >1 ectoparasite or vectorborne pathogen, to ectoparasites or vectorborne parasites only, and to filarial parasites in dogs in different age classes (Appendix Figure 1). The finding of clinical signs (e.g., respiratory, lymph nodes, ocular, and skin abnormalities and increased body temperature) was statistically associated with the overall detection of/exposure to >1 parasite (Appendix Figure 2) and with ectoparasite infestation or detection of/exposure to vectorborne parasites in dogs (Appendix Figure 3). For cats, we found no association between age group and detection of parasites, whereas clinical signs (i.e., enlarged lymph nodes and skin abnormalities) were statistically associated with detection of ectoparasitic infestation (Appendix Figure 4). We found no statistical association between seropositivity for FIV antibodies and FeLV antigens and detection of ectoparasites, vectorborne pathogens, or both.

## Discussion

The detection of zoonotic pathogens in client-owned dogs and cats living in metropolitan areas indicates that these animals serve as hosts for several parasitic agents in Asia. We provide data for an extended geographic distribution of zoonotic pathogens (e.g., *L. infantum* protozoa and zoonotic species of filariae) and of arthropods infesting animals (e.g., ticks of the *Haemaphysalis* and *Rhipicephalus* genera) where prior data unavailability made treatment and disease control strategies unachievable.

Nearly half of the dogs and one third of the cats in this study were infested with >1 ectoparasite or exposed to vectorborne pathogens; prevalence peaked in countries with a humid tropical climate (e.g., the Philippines, where 67% of dogs were infested with ticks, and Malaysia, where 89% of cats were infested with fleas). Such findings raise concern that vectorborne pathogens are responsible for several zoonotic diseases in Southeast Asia (*25*). The most prevalent tick on dogs and cats in this study was *R. sanguineus.* The taxonomic status of this tick group is a matter of debate with regard to *R. sanguineus* sensu lato including 2 lineages, so-called temperate and tropical (*26**–**28*). The tropical lineage of the *R. sanguineus* s.l. tick is prevalent in most countries in Asia and has been deemed accountable for the transmission of pathogens causing babesiosis, ehrlichiosis, and several rickettsial diseases in Asia (*25*,*29*,*30*). Despite the high proportion of tick-infested animals, the paucity of data on the ecology of *R. sanguineus* s.l. ticks in Asia makes their role as a vector difficult to ascertain. 

Unexpectedly, we found tick species not classically associated with companion animals but with the potential to transmit zoonotic disease–causing pathogens in dogs. For example, *Haemaphysalis hystricis* ticks have been implicated as vectors of a novel *Borrelia* species closely related to the relapsing fever group (*31*), and *Haemaphysalis wellingtoni* ticks are vectors of Kyasanur Forest disease virus, which causes fatal epidemics among monkeys and leads to hospitalization of ≈500 persons/year in India (*3*). Moreover, dogs seropositive to *B. burgdorferi* s.l. in this study were from Indonesia and the Philippines. This finding is unexpected, considering that these bacteria have been detected outside the known distribution area of *Ixodes* tick species, the main vectors of *B. burgdorferi* s.l., and indicates a need for in-depth epidemiologic surveys of this group of pathogens in Southeast Asia.

These results update the list of pathogens and ectoparasites affecting companion animals in Asia, including ticks with multihost feeding behavior, which has the potential to extend the network of pathogen transmission further into urban areas. The same holds true for pet dogs, suggesting that these animals might have been overlooked as potential pathogen reservoirs in metropolitan settings in this geographic area.

Similarly, the *Ctenocephalides orientis* flea was identified in one fifth of flea-positive dogs. The host spectrum of this flea is wider, but apparently its geographic distribution is more limited than that of the well-known cat flea *Ctenocephalides felis*, and it is involved in the transmission of rickettsiae, including *Rickettsia* sp. genotype RF2125 and *Rickettsia* sp. TH2014 (*32*,*33*). The morphologic ambiguity of the *C. orientis* flea (probably misidentified as *Ctenocephalides canis* and previously reported as a subspecies of *C. felis*) has contributed to a substantial dearth of information on its global distribution and role as a vector. In contrast, the cosmopolitan *C. felis* flea has colonized different bioclimatic niches, mainly through human-mediated migration (*34*). As human and animal global transportation increase in Asia, constant vigilance regarding the introduction of *C. orientis* fleas outside their known range of distribution in developed and developing countries is essential, as supported by the recent report of detection of fleas of this species in Iran (*35*).

In Singapore, one of the countries with the highest human development index (*36*) and lowest proportion of animals affected by parasites, *Lynxacarus radovskyi*, a mite for which little is known regarding its ecology, was detected on 35% of sampled cats. We have provided molecular data and updated morphologic information on this listophorid mite, which is an agent of papular dermatitis in humans (*37*). Furthermore, availability of appropriate diagnostics for this species or data on the efficacy of ectoparasiticides against it are limited.

Refined diagnostics are essential for assessing the distribution of filarial species in canine populations. For instance, the poor agreement (κ *=* 0.271) between cPCR and antigen-detection tests for *D. immitis* advocates for the use of integrated diagnostics to better appreciate the epidemiologic status of this species of filariae. Furthermore, the use of both tests revealed *B. pahangi* and *B. malayi* to also (in addition to *D. immitis*) affect companion animals in the regions investigated. These 3 species of filariae cause clinical manifestations in humans: lymphatic filariosis for *B. malayi* and *B. pahangi* (*38*,*39*) and pulmonary granulomas for *D. immitis* (*40*). In particular, lymphatic filariosis is among the most debilitating neglected tropical diseases; an estimated 70 million persons are infected, among which >50% live in Southeast Asia (*41*,*42*), and *D. immitis* infection of humans poses significant diagnostic challenges (*40*). Hence, for development and enactment of global elimination programs (*41*), surveillance of filarial species should be extended to animal populations in filariae-endemic countries (*42*).

Similarly, *Leishmania* spp. parasites currently cause ≈500,000 human infections/year in 62 countries (*43*), although their occurrence in eastern and Southeast Asia is poorly documented. We detected dogs positive for *L. infantum* by serology, qPCR, and sequencing in China and seropositive dogs in Thailand and Vietnam. In Thailand, the recent emergence of *L. martiniquensis* and *L. siamensis* caused immunocompetent and immunocompromised persons to seek medical assistance (*44*). A range of animals is involved in the zoonotic cycle of these 2 species (*44*,*45*), but dogs are the main reservoir for zoonotic leishmaniosis caused by *L. infantum* (*46*). The role of *Leishmania* spp. in human infections and as agents of disease in Southeast Asia requires urgent attention.

Further complicating knowledge of the transmission of zoonotic parasites in these regions of Asia are the large populations of free-roaming animals; the increased number of pet dogs and cats; and the complex social, economic, and ecologic changes currently occurring in Asia, (*1*,*2*,*4*,*25*,*47*,*48*). Integrated strategies that address all of these factors are therefore fundamental for the control of such parasitic agents. We investigated the presence of pathogens and ectoparasites in pet dogs and cats living in metropolitan areas in close proximity to humans. These animals share a common environment with humans, which makes them likely key reservoirs for pathogens with the potential to infect persons living in such areas and settings.

The epidemiologic data presented in this study can be pivotal for building knowledge bases about the occurrence of zoonotic parasites infecting companion dogs and cats in eastern and Southeast Asia. This information could help epidemiologists and policy makers provide tailored recommendations in the blueprint of future surveillance and prevention strategies.

Appendix Supplementary methods and results for study of ectoparasites and vectorborne zoonotic pathogens of dogs and cats from eastern and Southeast Asia, 2017–2018.
