# Supplementary material for: Zoonotic Vectorborne Pathogens and Ectoparasites of Dogs and Cats in Eastern and Southeast Asia
Source: Emerg Infect Dis. 2020 Jun;26(6):1221–33. doi: 10.3201/eid2606.191832 (PMC7258489; doi:10.3201/eid2606.191832)
Supplement: Appendix — Supplementary methods and results for study of ectoparasites and vectorborne zoonotic pathogens of dogs and cats from eastern and Southeast Asia, 2017–2018. [file 19-1832-Techapp-s1.pdf]

Article DOI: https://doi.org/10.3201/eid2606.191832

Ectoparasites and Vectorborne Zoonotic Pathogens of Dogs and Cats in Eastern and Southeast Asia, 2017–2018

Appendix

Appendix Table. Target genes, primers (sequence and length) and cPCR/qPCR cycling conditions used in this study to detect and characterize parasites from dogs and cats

Species/Pathogen	Target gene	Primers	Sequence (5′-3′)	Fragment length (bp)	Reference
Nematodes	*cox*1	NTF<br>NTR	TGATTGGTGGTTTTGGTAA<br>ATAAGTACGAGTATCAATATC	648	(*1*)
Ticks	16S rRNA	RHS16SF<br>RHS16SR	CTGCTCAATGATTTTTTAAATTGCTGT<br>TTACGCTGTTATCCCTAGAG	300	(*2*)<br>Modified as follows: 94°C for 10 min initial denaturation, followed by 35 cycles at 94°C for 45s, 58°C for 45s, 72°C for 60s and 72°C for 7 min for the final elongation.
Fleas, lice, mites	*cox*1	LCO1490<br>HCO02198	GGTCAACAAATCATAAAGATATTGG<br>TAAACTTCAGGGTGACCAAAAAATCA	710	(*3*)<br>Modified as follows: 95°C for 10 min followed by 35 cycles at 95°C for 60 s, 44°C for 60 s, 72°C for 90 s, and 7 min at 72°C for final elongation.
Fleas	*cox*1	LCO1490<br>Cff-R	GGTCAACAAATCATAAAGATATTGG<br>GAAGGGTCAAAGAATGATGT	601	(*4*)<br>Modified as follows: denaturing at 95°C for 10 min followed by 35 cycles at 95°C for 30 s, 52°C for 30 s, 72°C for 45 s, and 7 5 min at 72°C for final elongation.
Notoedres/ *Lynxacarus*	18S rRNA	Mite18S-F<br>Mite18S-R	ATATTGGAGGGCAAGTCTGG<br>TGGCATCGTTTATGGTTAG	464–490	(*5*)
Babesia/ *Hepatozoon* spp.	18S rRNA	RLB-F<br>RLB-R	GAGGTAGTGACAAGAAATAACAATA<br>TCTTCGATCCCCTAACTTTC	460	(*6*)<br>Modified as follows: 95°C for 10 min initial denaturation, followed by 40 cycles at 95°C for 30s, 52°C for 30s, 72°C for 60s and 72°C for 7 min for the final elongation.
Leishmania spp.	ITS-2	LGITSF2<br>LGITSR2	GCATGCCATATTCTCAGTGTC<br>GGCCAACGCGAAGTTGAATTC	383–450	(*7*)<br>Modified as follows: 95°C for 10 min initial denaturation, followed by 35 cycles at 95°C for 30s, 60°C for 30s, 72°C for 60s and 72°C for 7 min for the final elongation.
Leishmania spp.	kDNA minicircle	MC-1<br>MC-2	GTTAGCCGATGGTGGTCTTG<br>CACCCATTTTTCCGATTTTG	447	(*8*)
Leishmania spp.	kDNA minicircle	LEISH-1<br>LEISH-2<br>Probe	AACTTTTCTGGTCCTCCGGGTAG<br>ACCCCCAGTTTCCCGCC<br>FAM-AAAAATGGGTGCAGAAAT	120	(*9*)

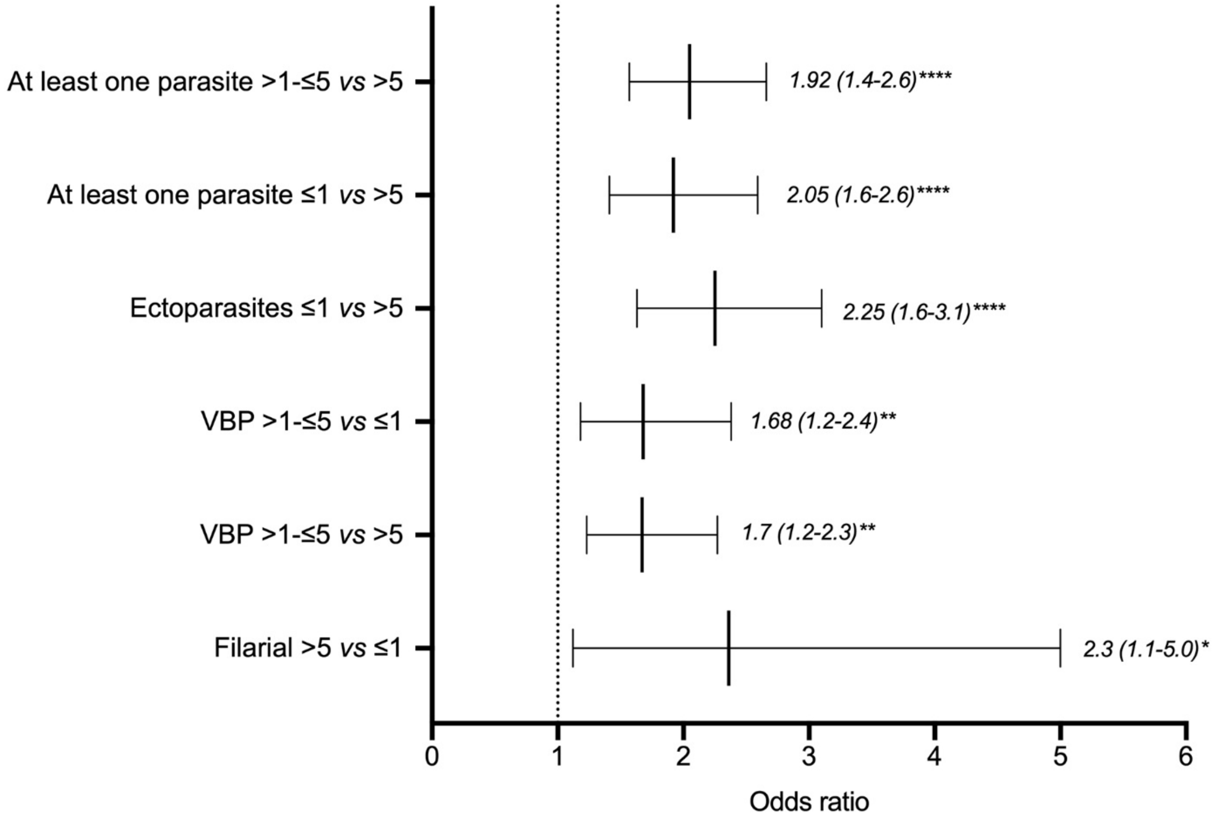

Appendix Figure 1. Box plot of odds ratio (median and range) of the detection/exposure to at least one vector-borne pathogen or ectoparasite, ectoparasites- or vector-borne pathogens-only, and to filarial parasites in dogs aging ≤1, >1-≤5 and >5 years. Odds ratio and CI 95% in brackets. **** $p < 0.0001$, ** $p < 0.001$, * $p < 0.01$, ns not significant.

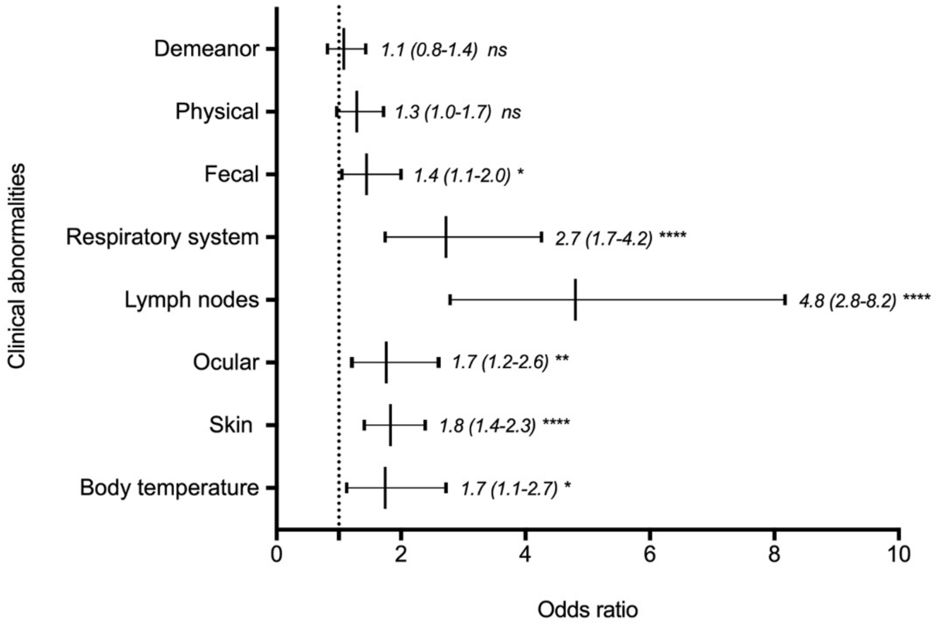

Appendix Figure 2. Box plot of odds ratio (median and range) of selected clinical abnormalities associated with overall detection/exposure to at least one parasite in dogs. Odds ratio and CI 95% in brackets. **** $p < 0.0001$, ** $p < 0.001$, * $p < 0.01$, ns not significant.

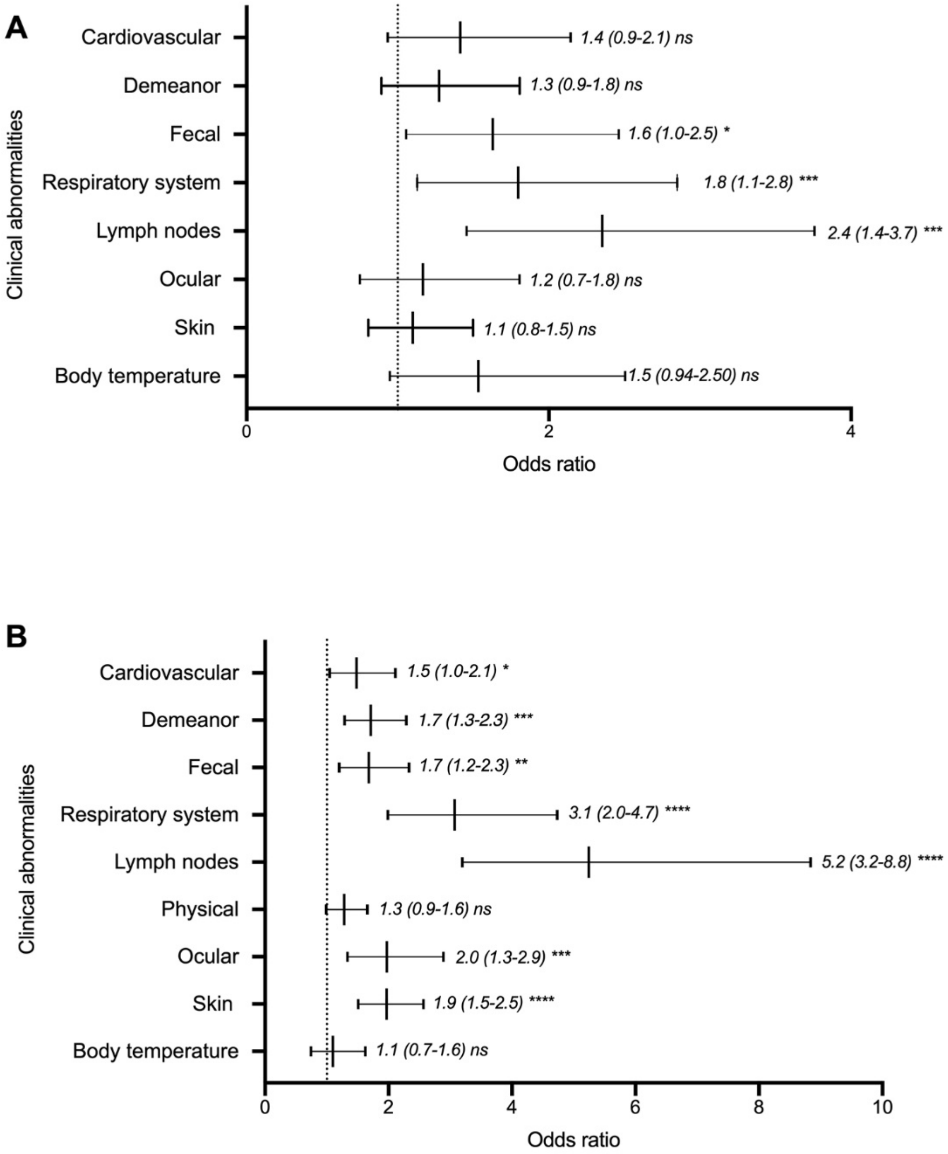

Appendix Figure 3. Box plot of odds ratio (median and range) of selected clinical abnormalities associated with the detection/exposure to VBPs (**A**) or ectoparasitic infestation in dogs (**B**). Odds ratio and CI 95% in brackets. **** $p < 0.0001$, *** $p < 0.001$, ** $p < 0.002$, * $p < 0.01$, ns not significant.

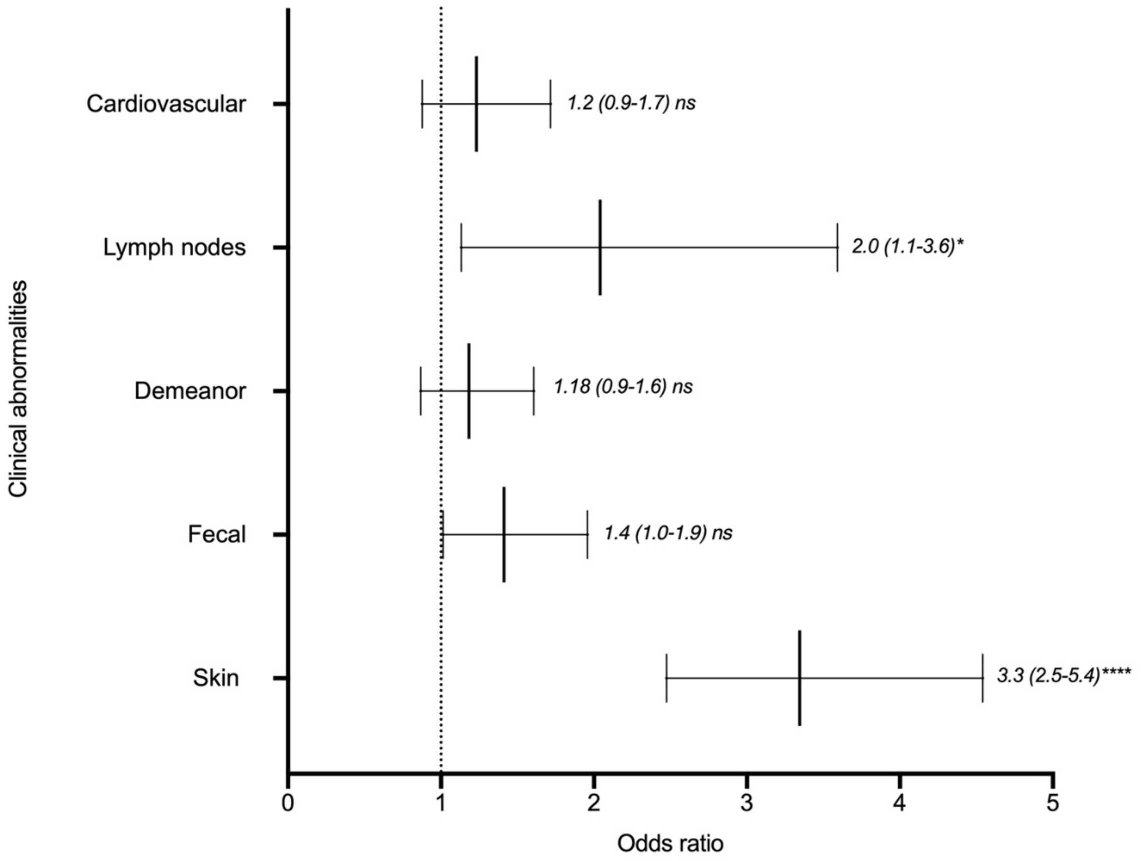

Appendix Figure 4. Box plot of odds ratio (median and range) of selected clinical abnormalities associated with the detection of ectoparasitic infestation in cats. Odds ratio and CI 95% in brackets. **** $p < 0.0001$, * $p < 0.01$, ns not significant.

References

1. Casiraghi M, Anderson TJ, Bandi C, Bazzocchi C, Genchi C. A phylogenetic analysis of filarial nematodes: comparison with the phylogeny of *Wolbachia* endosymbionts. Parasitology. 2001;122:93–103. PubMed https://doi.org/10.1017/S0031182000007149

2. Burlini L, Teixeira KRS, Szabó MPJ, Famadas KM. Molecular dissimilarities of *Rhipicephalus sanguineus* (Acari: Ixodidae) in Brazil and its relation with samples throughout the world: is there a geographical pattern? Exp Appl Acarol. 2010;50:361–74. PubMed https://doi.org/10.1007/s10493-009-9321-8

3. Folmer O, Black M, Hoeh W, Lutz R, Vrijenhoek R. DNA primers for amplification of mitochondrial cytochrome c oxidase subunit I from diverse metazoan invertebrates. Mol Mar Biol Biotechnol. 1994;3:294–9. PubMed

4. Lawrence AL, Brown GK, Peters B, Spielman DS, Morin-Adeline V, Šlapeta J. High phylogenetic diversity of the cat flea (*Ctenocephalides felis*) at two mitochondrial DNA markers. Med Vet Entomol. 2014;28:330–6. PubMed https://doi.org/10.1111/mve.12051

5. Sourassou NF, De Moraes GJ, Júnior ID, Corrêa AS. Phylogenetic analysis of *Ascidae* sensu lato and related groups (Acari: Mesostigmata: Gamasina) based on nuclear ribosomal DNA partial sequences. Syst Appl Acarol. 2015;20:225–40. https://doi.org/10.11158/saa.20.3.1

6. Gubbels JM, de Vos AP, van der Weide M, Viseras J, Schouls LM, de Vries E, et al. Simultaneous detection of bovine *Theileria* and *Babesia* species by reverse line blot hybridization. J Clin Microbiol. 1999;37:1782–9. PubMed https://doi.org/10.1128/JCM.37.6.1782-1789.1999

7. de Almeida ME, Steurer FJ, Koru O, Herwaldt BL, Pieniazek NJ, da Silva AJ. Identification of *Leishmania* spp. by molecular amplification and DNA sequencing analysis of a fragment of rRNA internal transcribed spacer 2. J Clin Microbiol. 2011;49:3143–9. PubMed https://doi.org/10.1128/JCM.01177-11

8. Cortes S, Rolão N, Ramada J, Campino L. PCR as a rapid and sensitive tool in the diagnosis of human and canine leishmaniasis using *Leishmania donovani* s.l.-specific kinetoplastid primers. Trans R Soc Trop Med Hyg. 2004;98:12–7. PubMed https://doi.org/10.1016/S0035-9203(03)00002-6

9. Francino O, Altet L, Sánchez-Robert E, Rodriguez A, Solano-Gallego L, Alberola J, et al. Advantages of real-time PCR assay for diagnosis and monitoring of canine leishmaniosis. Vet Parasitol. 2006;137:214–21. PubMed https://doi.org/10.1016/j.vetpar.2006.01.011
